# Simultaneous utilization of glucose and xylose for lipid accumulation in black soldier fly

**DOI:** 10.1186/s13068-015-0306-z

**Published:** 2015-08-14

**Authors:** Wu Li, Mingsun Li, Longyu Zheng, Yusheng Liu, Yanlin Zhang, Ziniu Yu, Zonghua Ma, Qing Li

**Affiliations:** College of Science, Huazhong Agricultural University, Wuhan, People’s Republic of China; State Key Laboratory of Agricultural Microbiology, Huazhong Agricultural University, Wuhan, People’s Republic of China; College of Engineering, Huazhong Agricultural University, Wuhan, People’s Republic of China; Institute of Environmental Biology and Insect Resources, College of Plant Protection, Shandong Agricultural University, Tai’an, People’s Republic of China

**Keywords:** Biodiesel, Rice straw, Glucose, Xylose, Black soldier fly

## Abstract

**Background:**

Lignocellulose is known to be an abundant source of glucose and xylose for biofuels. Yeasts can convert glucose into bioethanol. However, bioconversion of xylose by yeasts is not very efficient, to say nothing of the presence of both glucose and xylose. Efficient utilization of xylose is one of the critical factors for reducing the cost of biofuel from lignocelluloses. However, few natural microorganisms preferentially convert xylose to ethanol. The simultaneous utilization of both glucose and xylose is the pivotal goal in the production of biofuels.

**Results:**

In this paper, we found that 97.3 % of the glucose and 93.8 % of the xylose in our experiments was consumed by black soldier fly (BSF) simultaneously. The content of lipid reached its highest level (34.60 %) when 6 % xylose was added into the standard feed. 200 g of rice straw was pretreated with 1 % KOH, followed by enzymatic hydrolysis for fermentation of ethanol, the residue from this fermentation was then fed to BSF for lipid accumulation. In total, 10.9 g of bioethanol and 4.3 g of biodiesel were obtained.

**Conclusions:**

The results of this study suggest that BSF is a very promising organism for use in converting lignocellulose into lipid for biodiesel production.

**Electronic supplementary material:**

The online version of this article (doi:10.1186/s13068-015-0306-z) contains supplementary material, which is available to authorized users.

## Background

Lignocellulose is the most abundant and sustainable biomass on Earth, it can be used in the production of biofuels and other chemicals [1, 2]. The current bioethanol production processes typically involve 3 steps: physical or chemical pretreatment of biomass, enzymatic hydrolysis for saccharification, and fermentation for biofuel production [3, 4]. However, biofuel production remains extremely costly due to the costs of raw materials, pretreatment procedures, and management of environmental pollution which have prevented the large-scale production of biofuels in most regions [5, 6]. Thus, low-cost sources of raw materials must be identified to reduce costs.

Non-food feedstocks are frequently lauded as ideal carbon sources for biofuel production [7, 8]. Lignocellulosic biomass, with its high polysaccharide content, is abundant in nature and is well suited for biofuel production. Xylose accounts for as much as 35–45 % of the total sugar in lignocellulosic hydrolysates [9]. However, the bioconversion of xylose into ethanol is problematic. The simultaneous utilization of glucose and xylose is an important biochemical strategy that can be employed to improve the use of lignocellulosic biomass as a carbon source and thus reduce costs [10].

Bioethanol and biodiesel are two different examples of biofuels. Biodiesel provides substantial benefits over the more popular bioethanol and is considered the most attractive biofuel alternative to bioethanol. Nevertheless, the development and application of biodiesel has been hindered by the high cost of the required feedstocks [11]. To reduce these input costs in biodiesel production, the development of non-food biodiesel feedstocks is needed [12]. Due to this limitation, an alternative way of utilizing xylose needs to be found to increase the economic feasibility of biodiesel. Xylose is the second most abundant monosaccharide after glucose in the hydrolysates of lignocelluloses [13]. Efficient utilization of xylose in ethanol production is one of the critical obstacles in fuel ethanol production from lignocelluloses. Recently, the biofuel research community has focused increased attention on insects [14], particularly on scavenger species that have novel functions in lignocellulose digestion [15, 16].

Fortunately, many insects have shown the ability to efficiently degrade lignocellulosic substrates and to use these materials as a carbon supply [17]. Black soldier fly (BSF), *Hermetia illucens*, L., which can use different kinds of organic waste for lipid accumulation, is considered to be a promising non-food feedstock for biodiesel production [18, 19]. In previous studies, we demonstrated that *Boettcherisca peregrine* larvae and *Tenebrio molitor* L. could feed on organic wastes and could thus be important for biodiesel production from animal manures and food wastes; these studies did not examine xylose from lignocellulosic biomass [20, 21].

As organic wastes are not suitable for use in the human food chain but are highly abundant, organic wastes are considered to be relatively inexpensive feedstocks for biofuel. BSF is known to degrade organic wastes with high efficiency, and can colonize all kinds of organic wastes [22]. Thus, the use of BSF may make wastes profitable for use as feedstocks in biodiesel production. Some methods about the usage of BSF have already been developed, but they have not been found to be advantageous for production or economic perspectives. Therefore, this study was conducted to evaluate the use of lipid produced by BSF that consumed glucose and xylose from lignocelluloses for biodiesel.

## Methods

### Raw materials

BSF larvae were a gift from Dr. Jeffery K. Tomberlin of Texas A&M University, USA. The larvae were fed for about 6 days with standard colony diet before being used in this study. Rice straw was obtained from the Biomass and Bioenergy Research Center of Huazhong Agricultural University. The stalks were heated to 110–120 °C and held at this temperature for 10 min. After being dried, stalks were cut into small pieces and milled in a knife mill until the entire sample could pass through a 40 mesh screen.

### Glucose-induced lipid accumulation in BSF

Glucose and standard feed were mixed at various ratios to feed BSF for lipid accumulation. Based on preliminary trials, 6-day-old larvae (about 3 mg/larva) were inoculated into 1 g feed. To evaluate the effect of glucose on lipid yield, glucose and feed were mixed in the following ratios: 100 % feed (control), 1 % glucose, 2 % glucose, 4 % glucose, 6 % glucose and 8 % glucose. 1,000 of 6-day-old larvae were inoculated into 1,000 g mixed feed. The feeding experiments were terminated when prepupae accounted for most of the larvae in each treatment. The larvae were separated from the residual medium, and washed. Then they were killed at 110 °C for 10 min and dried at 60 °C until constant weights were obtained (no further reduction in weight with increased time). After being ground in a micro-mill, larvae powder was stored at 4 °C until the lipid extractions. All the experiments were carried out in a greenhouse at 27 °C with 70 % relative humidity.

### Xylose-induced lipid accumulation in BSF

Xylose and standard diet feed were mixed at various ratios to feed BSF for lipid accumulation. Based on preliminary trials, 6-day-old larvae (about 3 mg/larva) were inoculated into 1 g feed. The effect of xylose on lipid yield, which is known to be closely related to the final yield of biodiesel, was evaluated. Xylose and feed were mixed at the following ratios: 100 % feed (control), 1 % xylose, 2 % xylose, 4 % xylose, 6 % xylose, and 8 % xylose. 1,000 of 6-day-old larvae were inoculated into 1,000 g mixed feed. The feeding experiments were terminated when prepupae accounted for most of the larvae in each treatment. The protocols for the handling of the larvae in these experiments were the same as those described in the paragraph of glucose-induced lipid accumulation in BSF.

### Mixed xylose and glucose lipid accumulation in BSF

**Fig. 1:**
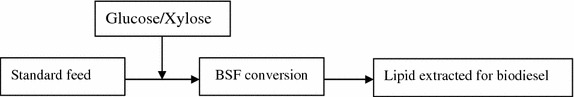
Diagram of bioconversion by BSF for biodiesel production from xylose.

Glucose, xylose and standard feed were mixed in various ratios to feed BSF for lipid accumulation. The process is shown in Fig. 1. Based on preliminary trials, 6-day-old larvae (about 3 mg/larva) were inoculated into 1 g feed. Glucose, xylose, and feed were mixed in the following ratios to evaluate the effect of combined glucose and xylose on the grease yield: a 100 % feed (control); B (6 % xylose + 6 % glucose). 1,000 of 6-day-old larvae were inoculated into 1,000 g mixed feed. The feeding experiments were terminated when prepupae accounted for most of the larvae in each treatment. The protocols for handling of the larvae in these experiments were the same as those described in the paragraph of glucose-induced lipid accumulation in BSF.

### Lipid accumulation in BSF induced by total sugar from rice straw

**Fig. 2:**
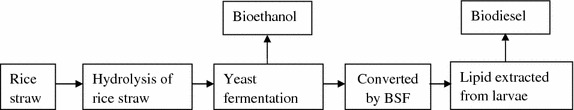
Diagram of bioconversion of BSF for bioethanol and biodiesel.

Figure 2 describes the bioconversion of BSF for bioethanol and biodiesel production from rice straw. 200.0 g of rice straw was mixed with 3,000 mL KOH (1.0 %, w/v) for 2 h, and the supernatant was collected. The residue was washed with distilled water and HAc–NaAc buffer (0.2 mol L^−1^, pH 4.8) and centrifuged at 3,000×*g* for 5 min. Multi-enzyme hydrolysate (Imperial Jade Bio-technology Co., Ltd. Ningxia, China) was then added for the hydrolysis, which was performed at 50 °C for 48 h with rotary shaking at 150 rpm. After hydrolysis, supernatants (hydrolysis and the KOH extracts) were combined and sterilized in an autoclave under 0.15 Mpa at 121 °C for 20 min. Fermentations were carried out using yeast for ethanol production (Angel yeast Co., Ltd., Yichang, China) in 5-L triangle bottles in an incubator at 37 °C for 48 h. The yeast powder was suspended in an appropriate amount of phosphate buffer (pH 4.8) to achieve 2.00 g L^−1^ yeast cell inoculum.

The fermentation liquid was distilled to collect the bioethanol. After that, 200 6-day-old BSF larvae were then inoculated into the residue from the fermentation for lipid accumulation. The feeding experiments were terminated when prepupae accounted for most of the larvae in each treatment. The protocols for the handling of the larvae in these experiments were the same as those described in the paragraph of glucose-induced lipid accumulation in BSF.

### Production of biodiesel

Lipid was obtained from each sample by the Soxhlet system using petroleum ether extraction twice for 8 h, petroleum ether was evaporated by a rotary evaporator, then lipid was calculated by weight. A two-step method was applied for biodiesel production, in which acid-catalyzed pretreatment (1 % H_2_SO_4_) was used as the first step to reduce the acid value and alkaline-catalyzed pretreatment (0.8 % KOH) was used for transesterification [18, 23].

### Analytical methods

Sugars were measured using colorimetric assays: glucose was measured with the anthrone/H_2_SO_4_ method [24], xylose was measured with the orcinol/HCL method [25]. A UV/VIS Spectrometer (Shanghai MAPADA Instruments Co., Ltd. V-1100D) was used for the glucose and xylose measurements. Hemicellulose was evaluated to measure the total amount of xylose, and cellulose content was evaluated to measure the total amount of glucose. The lipid was measured by weighing the samples before and after the extractions and taking the difference as the lipid value. Fatty acid composition was determined by GC/MC. All the experiments were carried out in triplicates.

### Statistical analysis

SPSS 17.0 (SPSS, Inc., Chicago, IL, USA) was used for statistical analyses. Correlations were analyzed using Spearman’s rank correlation analysis with a two-sided 0.05 level of significance (*P < 0.05, **P < 0.01). The variation and regression analysis were performed using Excel software. Differences among the samples were compared with Student’s *t* tests.

## Results and discussion

### Effects of glucose and feedstock ratios on BSF lipid content

Glucose is a primary energy source in many organisms, and can be converted into lipid. Excessive glucose in BSF is converted into lipid. In our study, lipid was extracted for biodiesel production. We observed that both the dry weight and the lipid content of BSF increased as the proportion of glucose in the feed increased (Additional file 1: Table S1).

BSF converted excessive glucose into lipid when additional glucose was added. The highest lipid content was 34.31 % of dry weight, when 8 % glucose was added, which was 7.78 % higher than that of the control (26.53 %). The lipid content reached 34.17 % of dry weight when the glucose concentration was 6 %, a value noticeably similar to that of the 8 % glucose treatment. Based on cost considerations, we concluded that the appropriate proportion of glucose in feed was 6 %.

### Effects of xylose and feedstock ratios on BSF lipid content

The accumulation of lipid in BSF changed with the ratio of xylose and standard feed which is shown in Additional file 1: Table S2. Different concentrations of xylose had a significant influence on the BSF dry weight and BSF lipid content. A gradual increase in the dry weight and lipid content of BSF was observed as the proportion of xylose increased. The maximum proportion of lipid observed in the xylose treatments was 34.60 %, while it was 26.85 % in the control group. The lipid content reached its highest level when the concentration of xylose was 6 %. Therefore, we concluded that the optimum proportion of xylose for lipid production was 6 %.

Sugar is an important platform organism for biological conversion of renewable biomass feedstocks into fuels and chemicals. Xylose is the second most abundant carbohydrate derived from plant materials, mainly hemicellulose hydrolysates from corn stalks, which are extremely rich in xylose. Although it is thought that yeasts cannot ferment xylose to ethanol, xylose can produce other economic products such as lipids. The results of our feeding experiments show that xylose can increase lipid content in BSF.

### Effects of mixed xylose and glucose on BSF lipid content

**Fig. 3:**
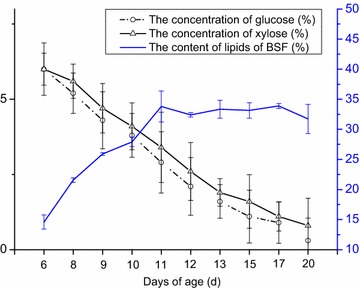
Dynamic changes of lipid content with mixed xylose and glucose.

Different ratios of xylose and glucose had a significant influence on the BSF dry weight and BSF lipid content. The mixed glucose and xylose led to significantly higher lipid content in BSF than standard feed when 6 % glucose and 6 % xylose were added (Fig. 3). BSF grew faster from the 6th to the 11th day than later days, and the content of lipid increased rapidly. The biomass and the content of lipid reached a stationary phase from the 11th to the 17th day. Afterwards, a gradual decline in lipid content was detected over the last 3 days.

The relationship between BSF lipid accumulation and sugar utilization was investigated and the results showed that BSF could consume glucose and xylose simultaneously for lipid accumulation without a time lag. Both glucose and xylose can act as substrates and be transformed into lipid stored in BSF.

Glucose decreased from 6 to 0.3 %, and xylose decreased from 6 to 0.8 %, while the lipid of larvae increased from 14.58 % up to 33.78 %. These results revealed dynamic changes in the glucose, xylose, and lipid contents in BSF: 97.3 % of the glucose and 93.8 % of the xylose were converted into lipid and BSF biomass within 14 days.

### Bioconversion of total sugar from rice straw by BSF

After pretreatment with 3,000 mL KOH, a multi-enzyme hydrolysate was added for the hydrolysis of rice straw. With the aid of pretreatment and digestion of cellulose, about 79.2 % of the cellulose and 85.7 % of hemicellulose could be hydrolyzed into sugars.

After the hydrolysis, the supernatant containing glucose (39.7 g) and xylose (25.9 g) was combined and then sterilized. Fermentations were carried out using *Saccharomyces cerevisiae* for ethanol production. The yield from the fermentation liquid was 10.9 g of ethanol. However, the bioconversion of xylose to bioethanol is not very efficient in terms of ethanol yield, most organisms prefer to use glucose over other monomeric sugars and do not assimilate other sugars until all of the glucose is consumed.

The residues from the fermentation above were then mixed with enzymatic hydrolyzed residues to feed the BSF larvae for lipid accumulation. Lignocelluloses, proteins, and reducing sugars were the main components of these residues. The fermentation residues mainly provided proteins while the enzymatic hydrolyzed residues provided lignocelluloses and other sugar sources. BSF can excrete cellulases for converting lignocelluloses, lipases for converting fats, oils, and proteases for converting proteins [19, 20, 23]. After 14 days of bioconversion, main components of the original mixed medium and the digested residuals were analyzed to compare the conversion rates of these components. BSF could convert about 89.6 % of the protein in the mixed feed. The constituents were converted by BSF for lipid bioconversion.

**Fig. 4:**
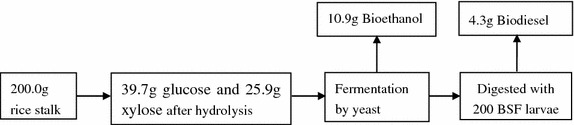
Bioconversion of lignocelluloses to bioethanol and biodiesel.

200 6-day-old BSF larvae (about 3 mg/larva) were inoculated into the mixture residues. Glucose and xylose were the major sugars in the hydrolysates of lignocellulosic biomass. BSF consumed glucose and xylose simultaneously. These sugars were converted into lipid in BSF. 5.2 g of total lipid was extracted by petroleum ether, and 4.3 g of biodiesel was obtained in the end. The diagram of the bioconversion progress is shown in Fig. 4.

Although the bioconversion of lignocellulosic residues into ethanol or butanol has been performed successfully [26, 27], biofuel production from cellulosic biomass remains a challenge, both technically and economically. These technologies are still under development. Therefore, recent research has been focused on the use of waste materials as renewable feedstocks to lower the cost of producing biodiesel. A large variety of agro-industrial residues have been used as prospective nutritional sources for BSF cultures. BSF can use lignocellulosic biomass for growth effectively and is thus considered as a promising alternative feedstock for biodiesel production.

The inability of many microbes to metabolize the xylose that is abundant within hemicellulose poses challenges for microbial biofuel production from cellulosic material; the obstacles associated with the use of lignocellulosic biomass have been explained [6]. The use of BSF to produce lipid from lignocellulosic biomass could be a potentially feasible approach for overcoming the obstacles that currently preclude the commercial production of biodiesel. We show that composition of rice straw can be utilized by BSF. Rice straw has high cellulose and hemicelluloses content that can be hydrolyzed into fermentable sugars. Utilization of glucose and xylose from organic wastes could be a feasible non-food feedstock for biodiesel.

### Fatty acid composition of BSF lipid from rice straw

In this study, BSF was investigated for its utility in biodiesel production from agriculture residues. H_2_SO_4_-catalyzed pretreatment as the first step for the reduction of acid value and KOH-catalyzed transesterification were used for biodiesel production. The composition of fatty acid methyl esters in the BSF biodiesel were lauric (29.8 %), palmitic (19.2 %), stearic (7.9 %), and oleic (28.8 %) acids; these accounted for over 85.7 % of the total fatty acids. Slight differences were found in various samples in terms of the relative fatty acid content in the fat products, regardless of the feedstock sources. The fatty acid composition of the BSF biodiesel is similar to that of biodiesels produced from vegetable oil, suggesting that insect lipid are a promising alternative source of lipid for biodiesel production [19].

## Conclusions

Effective utilization of glucose and xylose remains a big challenge for microbial bioconversions in biofuel production. Here, we show that BSF can consume xylose and produce lipid. BSF is thus a very promising organism for use to convert xylose into a valuable commodity. The accumulation of lipid for biodiesel from glucose and xylose is a promising alternative strategy for biodiesel production. The bioconversion of xylose is essential for the economic conversion of lignocellulose into biofuels. In this study, after 14 days of incubation, glucose and xylose were consumed by BSF. This study confirmed that BSF could convert lignocellulosic biomass into clean energy feedstocks and might be potential as a source of protein and organic fertilizer in future. In addition, the most abundant residues from agricultural crops have been given top priority as potential sources of biomass for biodiesel production. BSF is highly promising for use in converting xylose into an alternative feedstock for biodiesel, due to its high lipid content and short reproduction cycles.

## Additional file

Additional file 1:
**Table S1.** Effect of glucose on lipid accumulation in BSF (n = 3). **Table S2.** Effect of xylose on lipid accumulation in BSF (n = 3).
