# Supplementary material for: Simultaneous utilization of glucose and xylose for lipid accumulation in black soldier fly
Source: Biotechnol Biofuels. 2015 Aug 14;8:117. doi: 10.1186/s13068-015-0306-z (PMC4535370; doi:10.1186/s13068-015-0306-z)
Supplement: Additional file 1: — Table S1. Effect of glucose on lipid accumulation in BSF (n = 3). Table S2. Effect of xylose on lipid accumulation in BSF (n = 3). [file 13068_2015_306_MOESM1_ESM.docx]

**Table1 Effect of glucose on lipid accumulation in BSF (n=3)**

| **Proportion of glucose (%)** | **Dry weight (g/200 BSF)** | **lipid content (g/200 BSF)** |
| --- | --- | --- |
| Control | 10.03±0.51d | 26.53±1.03e |
| 1% | 11.41±0.17c | 28.16±0.28d |
| 2% | 12.49±0.26b | 31.06±1.17c |
| 4% | 13.07±0.77b | 33.35±0.72b |
| 6% | 15.66±0.41a | 34.17±0.88a |
| 8% | 16.32±0.64a | 34.31±0.82a |

Significant differences in BSF dry weights (df =5，F =19.354，p = 0.000) and lipid content (df=5，F=25.727，*p*=0.000) were found under different glucose concentrations, suggesting that increased glucose concentrations can increase BSF lipid content.

**Table 2 Effect of xylose on lipid accumulation in BSF (n=3)**

| **Proportion of xylose (%)** | **Dry weight (g/200 BSF)** | **lipid content (g/200 BSF)** |
| --- | --- | --- |
| Control | 8.64±0.20a | 26.85±1.94a |
| 1% | 10.42±0.60b | 29.36±1.00a |
| 2% | 10.02±0.36b | 29.91±1.36a |
| 4% | 11.71±0.42c | 33.64±0.12b |
| 6% | 11.59±0.10c | 34.60±0.89b |
| 8% | 12.02±0.51c | 34.24±0.34b |

According to variance analysis results, significant differences in BSF dry weights (df=5, F=43.032, p=0.000) and lipid content (df=5, F=24.156, p=0.000) were observed treatment with different proportions of xylose.
